# Heat Shock Protein 90 Chaperones E1A Early Protein of Adenovirus 5 and Is Essential for Replication of the Virus

**DOI:** 10.3390/ijms22042020

**Published:** 2021-02-18

**Authors:** Iga Dalidowska, Olga Gazi, Dorota Sulejczak, Maciej Przybylski, Pawel Bieganowski

**Affiliations:** 1 Department of Experimental Pharmacology, Mossakowski Medical Research Institute, Polish Academy of Sciences, Pawinskiego 5, 02-106 Warsaw, Poland; idalidowska@imdik.pan.pl (I.D.); dots@op.pl (D.S.); 2 Chair and Department of Medical Microbiology, Medical University of Warsaw, 02-091 Warsaw, Poland; olga.gazi@wum.edu.pl (O.G.); maciej@conexion.pl (M.P.)

**Keywords:** adenovirus, Hsp90, inhibitor, 17-AAG, E1A

## Abstract

Adenovirus infections tend to be mild, but they may pose a serious threat for young and immunocompromised individuals. The treatment is complicated because there are no approved safe and specific drugs for adenovirus infections. Here, we present evidence that 17-(Allylamino)-17-demethoxygeldanamycin (17-AAG), an inhibitor of Hsp90 chaperone, decreases the rate of human adenovirus 5 (HAdV-5) replication in cell cultures by 95%. 17-AAG inhibited the transcription of early and late genes of HAdV-5, replication of viral DNA, and expression of viral proteins. 6 h after infection, Hsp90 inhibition results in a 6.3-fold reduction of the newly synthesized E1A protein level without a decrease in the E1A mRNA level. However, the Hsp90 inhibition does not increase the decay rate of the E1A protein that was constitutively expressed in the cell before exposure to the inhibitor. The co-immunoprecipitation proved that E1A protein interacted with Hsp90. Altogether, the presented results show, for the first time. that Hsp90 chaperones newly synthesized, but not mature, E1A protein. Because E1A serves as a transcriptional co-activator of adenovirus early genes, the anti-adenoviral activity of the Hsp90 inhibitor might be explained by the decreased E1A level.

## 1. Introduction

Cytoplasmic heat shock protein 90 (Hsp90), which is an indispensable component of every eukaryotic cell, is represented in vertebrate cells by two closely related proteins, called Hsp90α and Hsp90β. Hsp90 is involved in the chaperoning of 200–300 clients proteins, and it is among the most abundant proteins in the cell [1,2]. Hsp90 clients include many proteins important for the regulation of cellular processes, such as hormone receptors, transcription factors, and protein kinases [3]. Many of the Hsp90 clients are involved in the development and progression of cancers, which makes this protein an attractive target for pharmacological intervention. The chaperoning activity of Hsp90 depends on the binding and hydrolysis of ATP by the N-terminal domain of this protein. Most of the identified inhibitors of Hsp90 binds to the ATP-binding pocket of ATPase. One of the first ATPase inhibitors of Hsp90 successfully used to inhibit Hsp90 chaperone activity in cultured mammalian cells was 17-N-allylamino-17-demethoxygeldanamycin (17-AAG) [4]. 18 Hsp90 inhibitors reached different stages of more than 170 clinical trials as a potential drugs in humans [5,6]. The dose-limiting toxicity was a major problem reported for the compounds tested so far. No clinical trials of the antiviral activity of the Hsp90 inhibitors have been conducted so far. The Hsp90 inhibitors interfered with the replication of many viruses that were tested in vitro, indicating that it might be possible to fight viral infections through Hsp90 inhibition. Interestingly, viruses tested so far appear to be sensitive to the non-toxic doses of Hsp90 inhibitors [7,8]. The replication of a virus requires the production of a large quantity of several types of proteins. These proteins often require help from cellular chaperones in proper folding and protection from aggregation. Hsp90 is involved in the replication of many viruses at different stages of the replication cycle by facilitating virus particle entry in the cell, intracellular transport, expression, and the stabilization of viral proteins, and genome replication [9,10,11]. Hsp90 may also be required for the virus assembly and trafficking [12,13]. Interestingly, the requirement for Hsp90 chaperone seems to be universal for the replication of viruses that belong to different taxonomic groups, but the function of Hsp90 seems to be specific for each virus [14,15,16].

Human adenoviruses (HAdV) belong to the *Adenoviridae* family and they are classified in the *Mastadenovirus* genus. Non-enveloped icosahedral virions of human adenoviruses are 70 to 90 nm in diameter with over 30 proteins encoded in a 35 kbp long double-stranded DNA. Human adenoviruses are divided into seven species (*Human mastadenovirus A-G*) with best-studied HAdV-2 and HAdV-5, both belonging to the species C [17,18,19]. Adenovirus infection is mostly associated with respiratory tract disease and conjunctivitis, while gastrointestinal and urinary tract disease less commonly occurs. Primarily associated with both occasional cases and epidemic infections among children, HAdVs now emerged as opportunistic pathogens causing significant morbidity and mortality in the immunocompromised population [20,21]. In immunocompetent hosts, adenovirus infections are usually mild and self-limiting, with the rare need for medical intervention.

HAdV-5 infection begins with the virus binding to the cell membrane, through the interaction of fiber protein with Coxsackievirus–adenovirus receptor (CAR), CD46, or sialic acid [22,23,24,25]. Subsequently, the penton base protein binds to integrins from α_v_β family that serve as an entry receptor and the virus is internalized in the endosomes by receptor-mediated endocytosis [26,27]. The transport of the viral DNA is usually completed in less than 1 h [28].

E1A RNA is the first to be transcribed after HAdV DNA enters the nucleus [29]. E1A proteins, which are translated from differentially spliced mRNAs, serve as co-activators of the remaining early promoters (E1B, E2A, E2B, E3, and E4) and regulate the transcription of many cellular genes [30]. E2 region encodes the proteins necessary for AdV DNA replication. E2A transcript translates to DNA-binding protein (DBP) and E2B transcript encodes polymerase and precursor terminal protein [31].

The subsequent transcription of the early AdV regions E3 and E4 results in the production of proteins active in inhibition of apoptosis and suppression of intracellular immune response and activation of the late promoter L4 [32,33,34,35,36,37].

DNA replication is initiated by the products of the E2 region. DNA replication also activates a transcription of the major late transcript from the late promoter. This transcript is alternatively spliced into several mRNAs that encode hexon, penton, fiber, and other structural proteins of the AdV capsid [38]. After the replication and capsids assembly is completed, virus is released by cell lysis [39,40].

HAdV infection leads to increased transcription of *HSP27*, *HSP70*, and *HSP90* genes [41]. Hsp70 interacts with adenoviral capsid proteins [42,43]. However, the specific function of heat shock proteins in HAdV replication was not studied. Therefore, in the present work, we decided to investigate the possible role of Hsp90 in HAdV-5 replication.

## 2. Results

### 2.1. Hsp90 Is Necessary for Efficient HAdV-5 Replication

**Figure 1:**
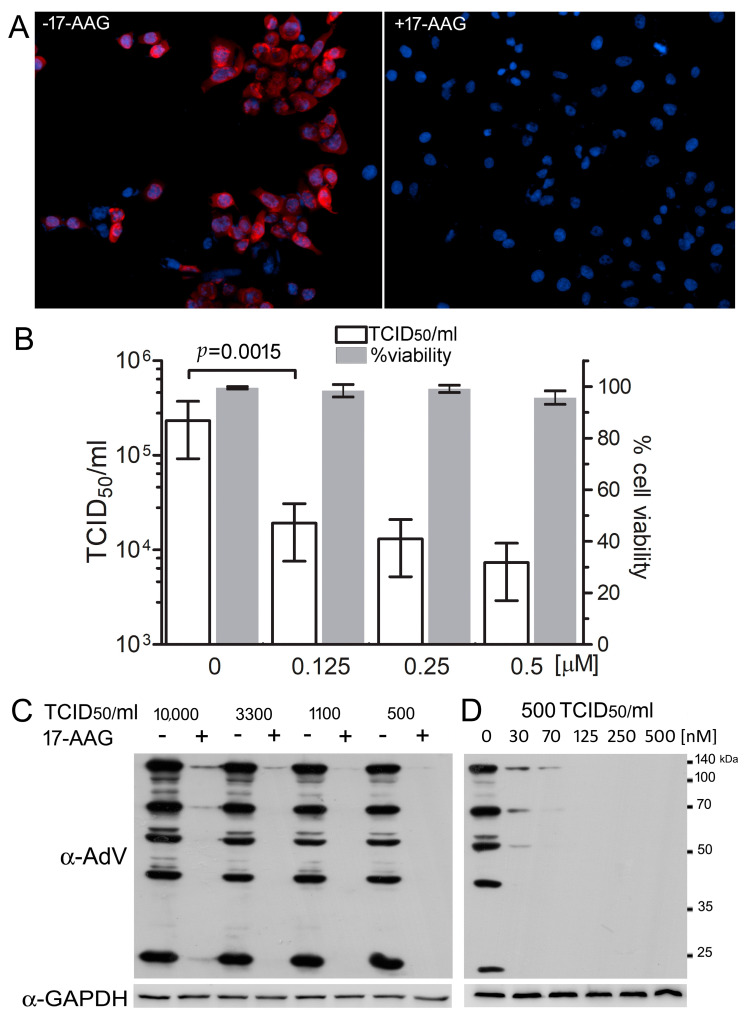
Hsp90 activity is necessary for AdV5 replication. (**A**) Cells A549 were infected with 500 TCID_50_/mL of AdV5 without 17-(Allylamino)-17-demethoxygeldanamycin (17-AAG) (panel A) or with 0.25 μM 17-AAG (panel B). Cells were stained with anti-human adenovirus 5 (HAdV-5) antibody (red) and DAPI (blue) 24 h after infection. (**B**) A549 cells infected with HAdV-5 were cultured for 48 h in the indicated concentrations of 17-AAG. The yield of the virus was measured using a cytopathic assay. Cell viability after 72 h of culture in the same 17-AAG concentrations was measured using MTT assay. Plotted are TCID_50_ and 95% confidence intervals values for the virus yield, and mean and SD values for the cell viability, and *p* value was calculated using one-tailed student’s *t*-test. (**C**) A549 cells infected with indicated concentration of HAdV-5 were cultured in 0.25 μM 17-AAG or without the inhibitor for 24 h. (**D**)A549 cells infected with 500 TCID_50_/mL of HAdV-5 were cultured in the indicated concentrations of 17-AAG for 24 h. Protein extracts were analyzed by western blot with anti-HAdV-5 antibody.

We used 17-AAG, a selective inhibitor of Hsp90 to test the role of Hsp90 in HAdV-5 replication. Human A549 cells were infected with the virus at 500 TCID_50_/mL in the presence of the inhibitor. Staining with a polyclonal antibody specific for the human HAdV-5 proteins demonstrated that, in the cells exposed to 0.5 μM 17-AAG, the expression of these proteins was not detectable 24 h after infection (Figure 1A). A cytopathic assay confirmed that, 48 h after infection, the yield of infective virus particles was 10 times lower in the presence of 0.125 μM 17-AAG, and 20 times lower in the presence of 0.5 μM 17-AAG, as compared to yield in control cultures without the inhibitor (Figure 1B). The results of the MTT assay demonstrated that over 95% of the cultured cells remained viable after 72 h, even at the highest, 0.5 μM concentration of the inhibitor, eliminating the possibility that the decreased rate of the virus replication may be attributed to the cytotoxic effect of 17-AAG. The 0.25 μM 17-AAG effectively inhibited the replication of the virus, even when the cells were infected with high doses of the virus (Figure 1C), and the inhibition was clearly visible, even in cultures with 0.03 μM 17-AAG (Figure 1D).

### 2.2. Hsp90 Inhibition Decreases Transcription of HAdV-5 Early and Late Genes and Replication of HAdV-5 Genome

**Figure 2:**
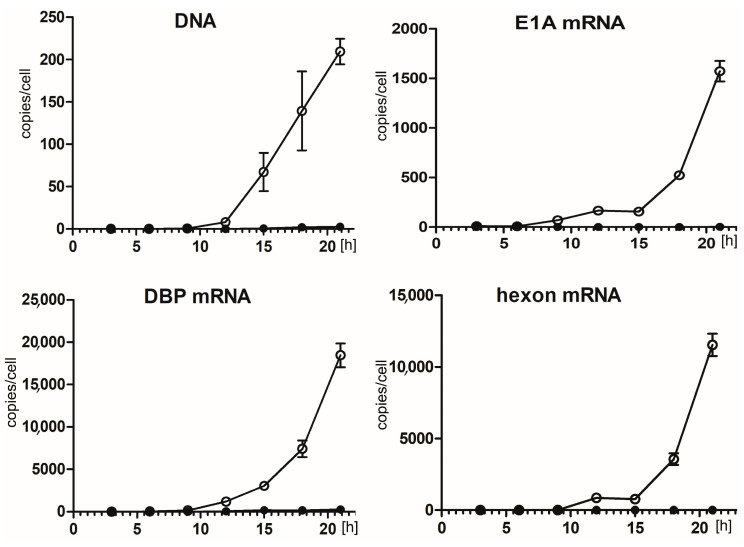
17-AAG inhibits transcription of AdV5 genes and replication of AdV5 genome. A549 cells infected with HAdV-5, were cultured in the presence of 0.25 μM 17-AAG, or without 17-AAG, for the specified time. HAdV-5 DNA and mRNAs were quantitated by qPCR. Plotted are numbers of DNA or mRNA copies per cell in cultures with 17-AAG (closed circles) and control cultures (open circles). Plots represent means and SD of the experiment performed in triplicate.

The results of the immunofluorescent staining and western blot analysis of the HAdV-5 infected cells demonstrated that 17-AAG inhibits the synthesis of the viral proteins. Therefore, we decided to test whether 17-AAG also inhibits the transcription and genome replication of HAdV-5. For that A549 cells were infected with HAdV-5 and cultured in a medium supplemented with 0.25 μM 17-AAG or without the inhibitor. Subsequently, HAdV-5 genomic DNA and transcripts for E1A, DBP, hexon were quantitated by qPCR. The exposure of cells to 17-AAG resulted in an 80-fold reduction of HAdV-5 DNA replication 21 h after infection. The effect of Hsp90 inhibition on mRNA was even greater. The number of transcripts was decreased over 800-fold for E1A and 8000-fold for hexon. Relatively lower, 70-fold reduction of DBP transcription corresponded well with the reduction in DNA replication (Figure 2).

### 2.3. 17-AAG Does Not Decrease the Level of Cellular Receptors for HAdV-5

**Figure 3:**
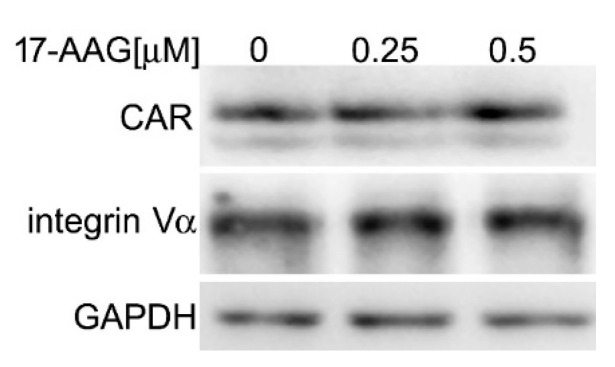
Level of receptors for HAdV-5 is not affected by Hsp90 inhibition. A549 cells were cultured for 24 h in the indicated concentrations of 17-AAG. 10 μg of a total protein extract was loaded on a gel and probed with antibodies specific for CAR and integrin αV. The experiment was repeated twice with identical results.

To test whether 17-AAG affects cellular receptors for HAdV-5, we compared expression of CAR and α_v_ integrin receptors in A549 cells after 24 h of treatment with 0.25 μM and 0.5 μM 17-AAG to the expression of CAR and α_v_ integrin receptors in cells that were cultured without the inhibitor. The unchanged amount of both proteins ruled out the possibility that the inhibition of AdV-5 replication is caused by the decreased availability of the receptors (Figure 3).

### 2.4. Expression of HAdV-5 Structural Proteins Is Sensitive to Hsp90 Inhibition Several Hours after Infection

**Figure 4:**
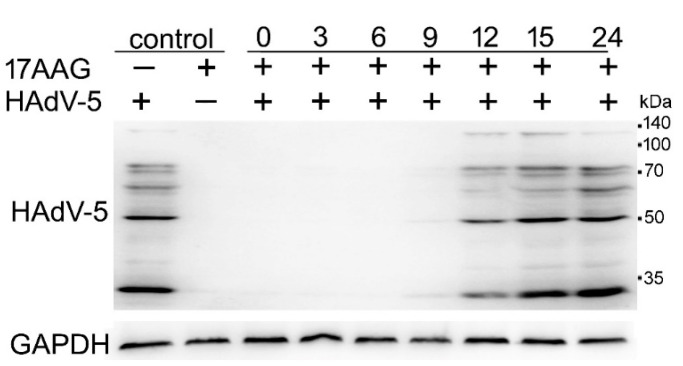
17-AAG inhibits translation of HAdV-5 proteins 9 h after infection. A549 cells were infected with HAdV-5. 17-AAG was added to a final 0.25 μM concentration at the indicated times and culture was continued to a total of 24 h. Expression of virus proteins was detected with anti-HAdV-5 polyclonal antibody.

We tested how the addition of the inhibitor at different time points after infection affects the expression of HAdV-5 proteins to elucidate which step of HAdV-5 replication is susceptible to 17-AAG presence. A549 cells that were infected with the virus were allowed to grow for the specified time. Subsequently, 17-AAG was added to a final 0.25 μM concentration, and incubation was continued for a total time of 24 h. All of the cell cultures were lysed 24 h after infection, and the protein extracts were analyzed by western blot with a polyclonal antibody that recognized several components of HAdV-5 capsid proteins. The results presented in Figure 4 demonstrate that Hsp90 inhibition substantially decreased the level of viral proteins, even when the inhibitor was added 9 h after infection, several hours after the DNA of the virus reached the nucleus, and after transcription and translation of the early genes began.

### 2.5. 17-AAG Inhibits Transcription of HAdV-5 Genes at the Early Steps after Infection

**Figure 5:**
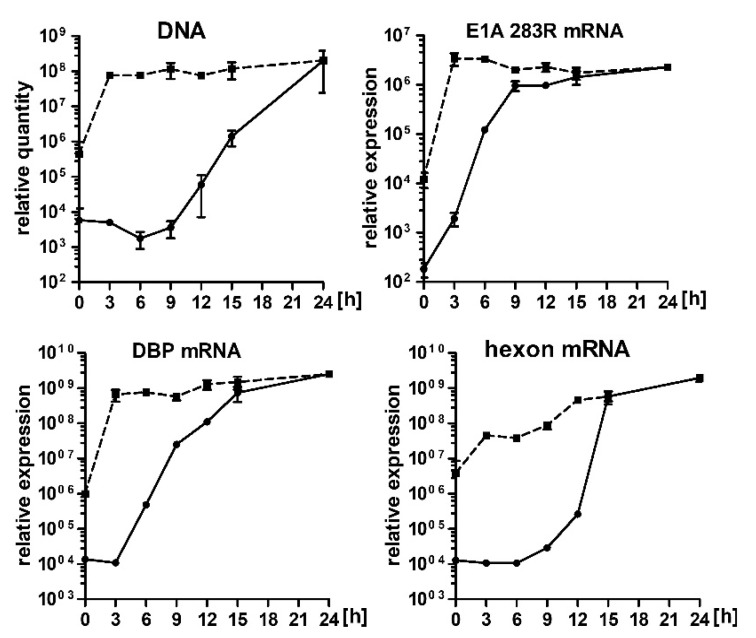
17-AAG inhibits HAdV-5 transcription and DNA replication early after infection. A549 cells were infected with HAdV-5. At the indicated times 17-AAG was added to a final 0.25 μM concentration (dotted line) or left without the inhibitor (solid line) and culture was prolonged to 24 h. 24 h after infection DNA and RNA was analyzed by q-PCR with primers specific for E1A, DBP, and hexon transcripts. DNA was analyzed using hexon-specific primers. The presented results are means and SD values from three experiments, normalized to results obtained for GAPDH.

0.25 μM 17-AAG was added to the infected cells at the specified time points and the expression of E1A, DBP, hexon mRNA, and the genomic DNA of the virus after 24 h of culture was measured by q-PCR in order to investigate if the decrease in the HAdV-5 protein levels described above resulted either from the reduced protein synthesis or the reduced transcription of the corresponding mRNAs. The mRNA expression of early genes (E1A and DBP) and the replication of the genomic DNA was only affected when 17-AAG was added immediately after infection. The hexone mRNA, expressed from the major late promoter was decreased in cells that were exposed to the inhibitor up to 9 h after infection (Figure 5).

### 2.6. Immediately after Infection 17-AAG Inhibits E1A Protein but Not E1A mRNA Expression

**Figure 6:**
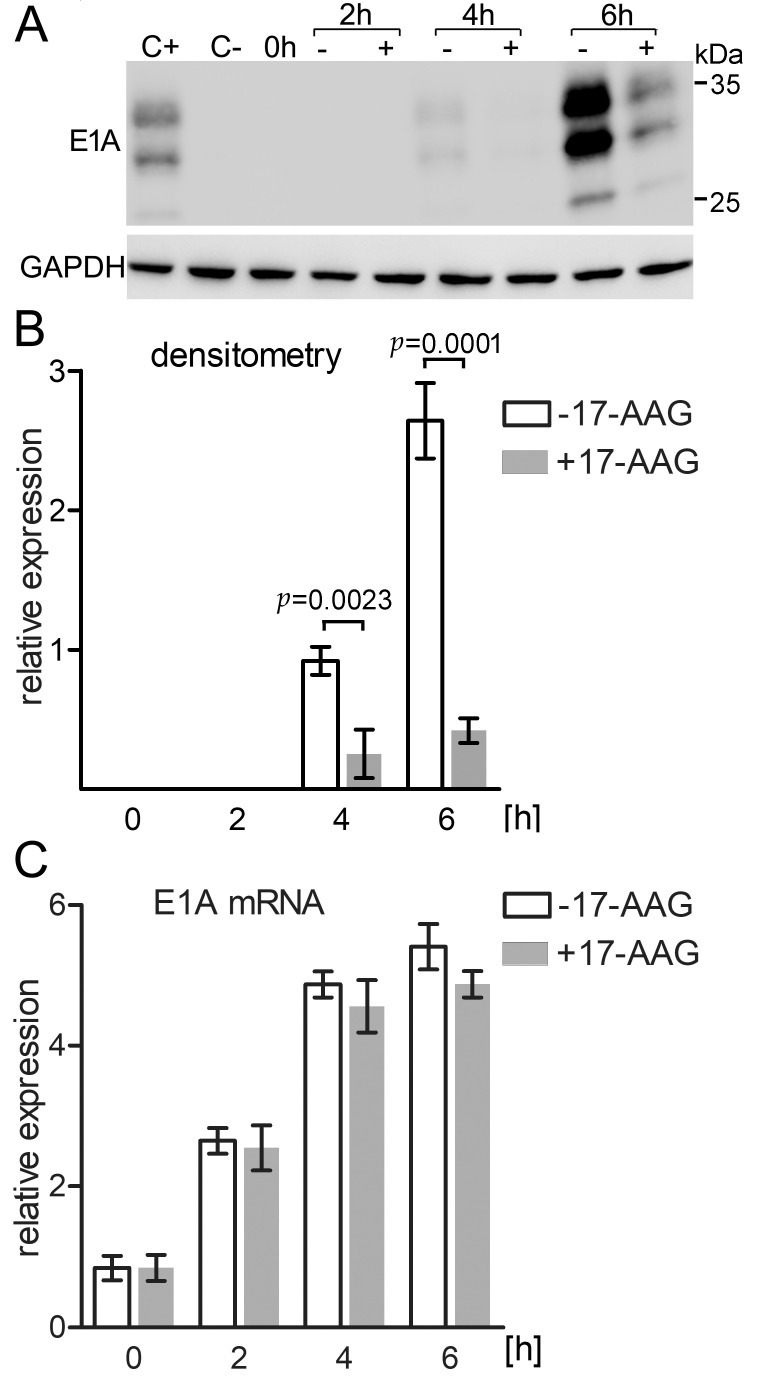
17-AAG inhibits E1A translation, but not transcription. (**A**) A549 cells were infected with HAdV-5 at TCiD_50_ 5 × 10^5^/mL for 15’ at 37 °C. After infection cells were washed 2 times with PBS and incubated in medium with 4 μM 17-AAG (+), or without it (-) for the indicated time. C- and C+ represent protein extracts of not infected and infected cells, respectively. Western blot was probed with E1A specific antibody. (**B**) Densitometric quantification of the western blot results for E1A normalized to GAPDH. (**C**) q-PCR was used to measure content of the E1A mRNA. q-PCR results were normalized to the results obtained with GAPDH specific primers. Plots B and C represent means and SD from three independent experiments.

Early E1A promoter is the first to be activated and the expression of the E1A protein is necessary for efficient transcription from other early promoters. The qPCR analysis demonstrated that E1A transcription is effectively inhibited by 17-AAG that is added to the culture at the time of infection but not 3 h later. Therefore, we decided to test how Hsp90 inhibition affects the expression of E1A mRNA and protein up to 6 h after HAdV-5 infection. A549 cells were infected with the virus at 1 × 10^5^ TCID_50_/mL for 15 min. in medium with 4 μM 17-AAG or without the inhibitor. The high concentration of the inhibitor was used to assure its effectiveness against the high titer of the virus combined with a short incubation time. After infection, cells were washed twice with PBS and the incubation in medium with or without 17-AAG was prolonged for 2, 4, and 6 h. The protein extract was analyzed by western blot with the E1A-specific antibody, and mRNA isolated from the cells was subjected to qPCR analysis with the E1A and GAPDH specific primers. The E1A protein was not detectable in control cells for the first 2 h after infection. 4 h after infection, Hsp90 inhibition resulted in a 3.6-fold reduction of the newly synthesized E1A protein level, and after 6 h the level of E1A in the control cells was 6.3-fold higher than in the cells that were treated with 17-AAG. These differences were statistically significant with *p* ≤ 0.0023 and *p* ≤ 0.0001, respectively (Figure 6A,B). E1A mRNA transcription was clearly detectable 2 h after infection, but the mRNA level did not change significantly in cells that were treated with 17-AAG when compared to control ones (Figure 6C). Together, these results demonstrate that Hsp90 inhibition affects the E1A protein level, but not its mRNA level.

### 2.7. The Inhibition of Hsp90 Increases Degradation Rate of the Newly Translated E1A

**Figure 7:**
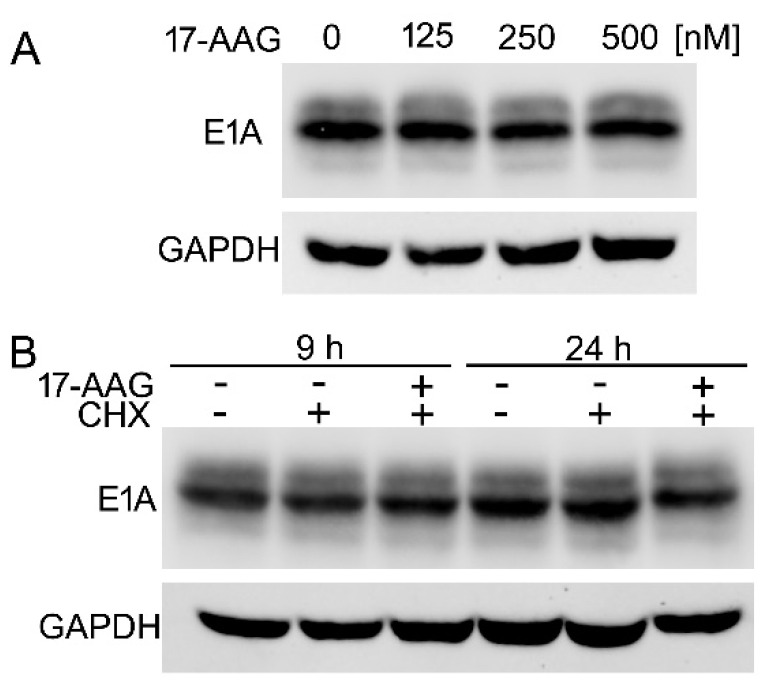
Hsp90 inhibition does not increase degradation of the mature E1A. (**A**) HEK293 cells were cultured for 24 h with the indicated concentrations of 17-AAG. (**B**) HEK293 cells were incubated with 500 nM 17-AAG, or with 500 nM 17-AAG and 100 µg/mL cycloheximide (CHX) for 9 and 24 h. Control cells were cultured without the inhibitors. E1A and GAPDH in the extracts were detected by western blot. Representative western blot, one of two performed is shown.

We analyzed the effect of the inhibitor on E1A protein expressed in HEK293 cell to test whether 17-AAG inhibits synthesis of E1A *de novo*, or increases the degradation rate of the E1A already present in the cells. These cells constitutively expressing the HAdV-5 E1A protein were clonally selected after transfection with a fragment of HAdV-5 DNA containing the E1A gene [44]. We found that the E1A protein remained stable in HEK293 cells that were exposed to 17-AAG for 24 h (Figure 7A). 17-AAG did not increase the rate of E1A protein decay. even when the protein synthesis was inhibited by cycloheximide (Figure 7B).

### 2.8. Hsp90α Interacts with E1A Protein

The E1A 289R (289 amino acid long product of the alternative splicing of E1A pre-mRNA) is the main transcriptional co-activator of the early promoters of HAdV-5. This form of E1A protein is both sufficient and necessary for the replication of the virus [45].

**Figure 8:**
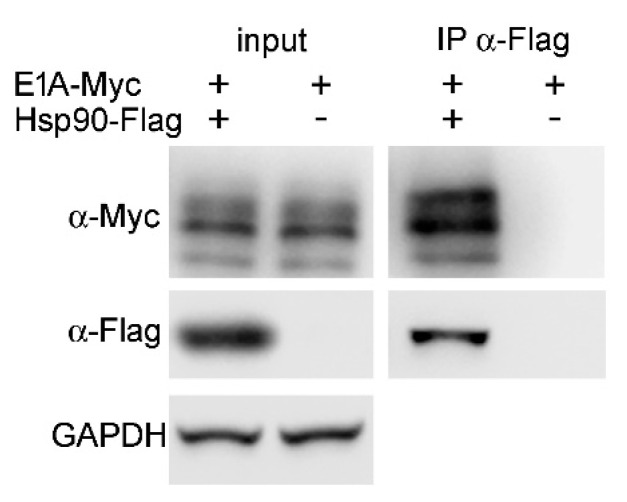
E1A 289R interacts with Hsp90α. HEK293 cells were transfected with plasmids that expressed E1A 289R-Myc and E46A mutant of Hsp90α-Flag as indicated. Flag specific antibody was used for immunoprecipitation. Cell extracts (input) and proteins bound to anti-Flag antibody (IP α-Flag) were probed with anti-Flag, or anti-Myc antibodies, to detect Hsp90α and E1A 289R respectively. Representative western blot, one of two performed is shown.

A co-immunoprecipitation assay was performed to test whether Hsp90 associates with E1A 289R [45]. Cells that were used in this assay were transfected with Flag-tagged Hsp90α E46A mutant and Myc-tagged E1A 289R. The complexes of Hsp90 with client proteins are usually unstable. Therefore, the E46A mutant of Hsp90 was used to stabilize its interaction with client proteins. The E46A substitution does not affect the ability of the mutant to bind client proteins, but the mutant lacks ATPase activity, necessary to complete the chaperoning cycle, and it remains in complex with a client protein. The results confirmed the association of Hsp90α and E1A 289R (Figure 8).

## 3. Discussion

Viruses utilize host cell cellular mechanisms to synthesize a large number of proteins that are involved in their replicative cycle. The inhibition of chaperoning activity of Hsp90 during infection prevents or suppresses the replication of many viruses that belong to different groups [46,47,48,49,50].

The aim of this study was to evaluate the effect of 17-AAG, the Hsp90 inhibitor, on human HAdV-5. We demonstrated that 17-AAG exerted a strong, concentration-depending, inhibitory effect on HAdV-5 replication at concentrations that did not affect cell viability. This effect was especially pronounced when the inhibitor was applied at the time of infection, which suggested that Hsp90 is required at the early steps of HAdV-5 replication.

Hsp90 inhibition does not influence the expression of the receptors that are necessary for HAdV-5 entry into the human mesothelioma JMN-1B cells [51]. We confirmed that this is also true for A549 cells, because, in 17-AAG treated cells, there was no decrease in the expression level of CAR and integrin α_v_, the receptors that are necessary for this process. Moreover, the synthesis of the viral proteins was inhibited by 17-AAG, even 9–12 h after infection, when the viral DNA reached the nucleus and transcription of the viral genes begun.

The time-course analysis of transcription revealed that 17-AAG inhibits the expression of the HAdV-5 early genes E1A and DBP at the time of infection, but it seems to be relatively ineffective when applied later. The expression of HAdV genes begins with the E1A transcription [52]. The E1A protein is necessary for the efficient transcription of other early HAdV-5 mRNAs and stimulates its own transcription [53]. Immediately after infection with the virus, the E1A transcription is catalyzed by the cellular proteins. Hsp90 inhibition did not affect this early transcription of the E1A gene, but the E1A protein level was decreased, which suggested that Hsp90 chaperones E1A protein. This conclusion was further supported by the Hsp90α-E1A association detected by co-immunoprecipitation. However, the E1A protein constitutively expressed in HEK 293 cells was not affected by the Hsp90 inhibition. Therefore, it seems that Hsp90 stabilized and protected from degradation the newly translated, but not the mature, E1A protein that was present in the cells before they were exposed to the inhibitor. The conclusion that 17-AAG affects only *de novo* expressed E1A protein was supported by the observation that 17-AAG did not increase the decay rate of E1A in HEK 293 cells after the protein synthesis was inhibited by cycloheximide.

Recently, the anti-HAdV activity of mifepristone was reported, attributed to the interference with steps preceding an entry of the virus genomic DNA into the nucleus [54]. The study of three salicylanilide anthelmintic drugs demonstrated that two of these compounds inhibit the HAdV life cycle by restricting access of the viral DNA to the nucleus, whereas the third one inhibited HAdV replication by decreasing E1A transcription [55]. All of these compounds were effective within 1 h after infection. The data presented here demonstrated that the Hsp90 inhibitor effectively limited HAdV-5 replication much later after infection. The expression of mRNAs for the proteins necessary for the viral genome replication, polymerase DBP, and PTP is activated by E1A. Therefore, the viral DNA replication depends indirectly on E1A. The decreased rate of the genome replication results in decreased production of the late viral proteins, not only due to the lower number of the gene copies, but also because the viral DNA replication activates the transcription of late promoter activator IVa [56].

The expression of capsid proteins, controlled by the late promoter, was especially sensitive to Hsp90 inhibition late after infection. These proteins are the last components of the virus to be synthesized. The expression of late mRNAs begins with the activation of the L4 promoter that drives the expression of L4-22K and L4-33K proteins [57]. These proteins are essential activators of the full set of late mRNAs [58]. L4 promoter is activated by the viral proteins E1A, E4 Orf3, and IVa2 [37]. The decreased E1A expression is a limiting factor for the viral capsid protein’s expression, not only directly, but also indirectly, because E1A also stimulates the expression of IVa2 and E4 orf3 [59,60].

A recently published study on HAdV inhibition by ivermectin, a drug preventing E1A protein from entering the nucleus, reports similar effects on the virus mRNA and protein expression and DNA replication, resulting in the decreased production of viral progeny similar to the reported here [61]. However, ivermectin inhibits E1A expression more effectively 24 h and 36 h after infection, whereas Hsp90 inhibition is most effective up to 9 h after infection.

Although there is a number of investigations focused on the role of Hsp90 in supporting viral replication, intracellular antiviral response, and virus trafficking, there are only limited data available concerning the chaperoning activity of Hsp90 for early viral activators of the replication process. Basha and collaborators presented an impact of geldanamycin (GA) on the expression of immediate early (IE) and major immediate early genes of human cytomegalovirus (CMV) [62]. There was a delay in IE2 (but not IE1) protein synthesis, and a significantly lower amount of IE2 was produced. The addition of GA at early steps of infection (0–8 hpi) lead to the effective inhibition of immediate early genes, which also led to a decreased synthesis of the second tier of major immediate early genes. Interestingly, following applications of GA doses during changes of cell culture medium led to the complete inhibition of CMV replication. Another work, by Katsuma, revealed the dependency of baculoviral IE protein on Hsp90 chaperone function [63]. Similar to our observations, treatment with 17-AAG did not affect the initiation of IE gene transcription, but it had a significant negative effect on stable IE protein synthesis. In both cases, conclusions indicated a fundamental role of Hsp90 in supporting viral replication via chaperoning of immediate early genes, while inhibition of this process resulted in disruption of viral gene expression cascade and eventually led to the delay or inhibition of the entire process of virus replication.

Contrary to the above-mentioned drugs that had anti-adenoviral activity, none of the Hsp90 inhibitors was approved for use in humans. This however may change with the new inhibitors being developed and numerous trials conducted. The data reported here demonstrated that 30 nM 17-AAG effectively inhibited HAdV-5 replication in vitro. 17-AAG antiviral activity at the non-toxic concentration was also reported in other studies [64,65,66]. The 17-AAG in plasma of the patients during the clinical trials reached 6–16 μM concentration, depending on the administered dose, with moderate adverse effects [67,68]. A lower concentration of the drug necessary to suppress viral infection may limit its toxic side effects. Antiviral drugs tend to lose effectiveness due to drug-resistant mutations. This may not be the case for the Hsp90 inhibitors, because a protein that depends on the Hsp90 chaperone for maturation and stability is not likely to be converted to the chaperone-independent and still functional variant by the simple mutation. There are known Hsp90 mutations that are resistant to ATPase inhibitors, but such mutations might occur in a limited number of cells, and they would not have an impact on the viral infection progress at the whole organism level [69].

Modified adenoviruses are widely used as vectors for DNA delivery into mammalian cells and HAdV modified to target tumor cells are studied as a potential means to treat cancers [70,71,72]. The applications of Hsp90 inhibitors in cancer treatment are also studied. The possible interference between the clinical application of Hsp90 inhibitors and HAdV-based agents should be considered. The data presented here suggest that replication-competent oncolytic adenoviruses may be particularly sensitive to the adverse effects of the Hsp90 inhibitors. The HAdV infections tend to be mild, but they can be serious in young and immunocompromised individuals, and specific drugs to treat such infections are lacking [73,74,75]. Our results indicate that Hsp90 inhibitors could be used to suppress the adenoviral infection.

## 4. Materials and Methods

### 4.1. Cell Lines and Virus Infection

The human epithelial cell line derived from lung carcinoma (A549) and human embryonic kidney 293 cells (HEK293) were obtained from ATCC and grown in Iscove’s Modified Dulbecco’s Medium (IMDM) that was supplemented with 10% fetal bovine serum, 100 U of penicillin, and 100 µg of streptomycin/mL (Sigma). Transfections were performed using Metafectane (Biontex), as suggested by the manufacturer. Human adenovirus 5 (VR-5) was obtained from ATCC.

In order to determine the effect of 17-AAG on HAdV-5 replication, A549 0.8 × 10^6^ cells/well were seeded in a six-well plate and then infected with the virus at the indicated titer. The inhibitor was added at the specified time concerning infection. Cycloheximide was used at 100 μg/mL concentration and 17-AAG was 0.25 μM, unless specified otherwise.

The virus titer was measured by 50% tissue-culture infectivity endpoint (TCID_50_) method of Reed and Muench [76].

### 4.2. Cell Viability Assay

Cell viability was determined using the Cell Counting kit–8 (Sigma). The cells were seeded into a 96-well plate in IMDM medium with different concentration of 17-AAG (0, 0.125, 0.25, 0.5 μM). After 72 h, cell viability was measured according to manufacturer instructions.

### 4.3. Plasmid Construction

E1A 289R DNA was amplified by RT-PCR from a total RNA isolated from HAdV-5 infected A549 cells using RNAzol and then converted to cDNA with hexamer primers in a reaction with AMV transcriptase. Primers E1A289F and E1A289R were used in this reaction. The resulting DNA fragment was cloned in the plasmid pcDNA-Myc using Kpn I and Xho I restriction sites that were incorporated in the sequence of the primers. The resulting plasmid expresses E1A 289R protein with the C-terminal Myc-tag from CMV promoter.

A plasmid that expresses the Flag-tagged Hsp90α gene in human cells was described earlier [69]. The Hsp90α E46A mutation was generated by PCR mutagenesis

Supplementary Table S1 lists the primers used to construct plasmids.

### 4.4. Immunofluorescence Microscopy

Prior to infection, A549 cells were seeded on coverslips and cultured overnight to adhere. The medium was removed and replaced with IMDM containing 0.25 μM 17-AAG and with HAdV-5 at 500 TCID_50_/mL and cultured for 24 h followed by 4% formaldehyde fixation, 0.1% Triton X-100 permeabilization and blocking with PBS containing 3% bovine serum albumin (BSA). The fixed cells were then stained with an anti-HAdV-5 rabbit polyclonal antibody (Abcam). Goat anti-rabbit secondary IgG antibody conjugated with Alexa Fluor Plus 598 (Invitrogen) was then added and DAPI was used to stain the nuclei.

### 4.5. Co-Immunoprecipitation Assay (Co-IP)

HEK293 cells were transfected with E1A 289R-Myc and Hsp90α E46A plasmids, while the control cells were transfected with pcDNA-Myc and Hsp90α E46A plasmids. After 48 h, cells were harvested and lysed with IP buffer (0,25% Triton X-100, 10 mM Tris, 20 mM NaF, 100 mM, 10 mM β-glycerol phosphate, 2 mM sodium orthovanadate, 5 mM ATP, and protease inhibitors cocktail (Roche)). The lysates were cleared by centrifugation at 12,000× *g* for 15 min. at 4 °C. The protein concentration was measured using the BCA assay (Sigma), and adjusted with IP buffer to 1 mg/mL. 10 μL anti-Flag agarose beads (Pierce) were added to the supernatant (700 μL), and then incubated for 2 h at 4 °C with mixing. The immunoprecipitates were washed with ice-cold PBS four times and eluted with 40 μL 1× SDS PAGE Loading buffer. The samples were boiled for 10 min. and analyzed by western blot.

### 4.6. Western Blot Analysis

The proteins were extracted by lysis with RIPA buffer. Rabbit polyclonal antibody for the HAdV-5 (ab6982) and for E1A (ab204123) were obtained from Abcam. Monoclonal antibodies were purchased from: Flag (Sigma, F3165), Myc (Merck, MABE282), CAR (Cell Signaling, 5670S), and integrin Vα (Cell Signaling, 60896S). Secondary antibodies that were conjugated to Alexa488 (Invitrogen, A32723) and Alexa594 (Invitrogen, A32740) were obtained from Abcam. Goat anti-rabbit IgG-HRP and Goat anti-mouse IgG-HRP antibodies were obtained from Bio-Rad (cat. no. 170-6515 and 170-6516). Western blot and immunofluorescence staining were performed according to the standard protocols with the antibodies diluted, as recommended by manufacturers.

### 4.7. qPCR

2 μg of RNA extracted with RNAzol reagent (Sigma) was used for cDNA synthesis while using hexamer primers and AMV reverse transcriptase. After synthesis, polymerase was inactivated and the reaction mixture was diluted with nine volumes of water. 1 μL of the cDNA was used as a template in a 20 μL PCR reaction with primers specific for E1A, DBP, hexon, and GAPDH (listed in Supplementary Table S1). The cDNA obtained as described above was used as a template in a qPCR reaction with primers and TaqMan probes listed in Supplementary Table S1. The results were expressed as the relative copy number of HAdV-5 mRNA or DNA normalized to GAPDH and G6PD, and then for a number of cells used for RNA extraction. QPCR for the viral DNA was performed using primers Hexon-F, Hexon-R, and Hexon probe.

### 4.8. Statistical Analysis

The one-tailed Student’s *t*-test was used for data analysis, with the significance set at 0.05.

## Data Availability

The data that support the findings of this study are available from the corresponding author upon reasonable request.

## Supplementary Materials

The following are available online at https://www.mdpi.com/1422-0067/22/4/2020/s1, Table S1: Oligonucleotides used in this study.
