# Supplementary material for: Heat Shock Protein 90 Chaperones E1A Early Protein of Adenovirus 5 and Is Essential for Replication of the Virus
Source: Int J Mol Sci. 2021 Feb 18;22(4):2020. doi: 10.3390/ijms22042020 (PMC7921956; doi:10.3390/ijms22042020)
Supplement: Supplementary file 1 [file ijms-22-02020-s001.pdf]

Supplementary Materials

Supplementary Table 1

List of primers

Primers and probes for qPCR

Hexon-F: GGACGCCTCGGAGTACCTGAG

Hexon-R: ACAGTGGGGTTTCTGAACTTGTT

Hexon probe: CTGGTGCAGTTCGCCCGTGCC

DBP-F: TAATCAAGCATGGCAAAGGAG

DBP-R: 5'-AATTTCACTTTCCGCTTCG

DBP probe: TGCGGACGCAAGAGGAAGAG

E1A F: TTGTCATTATCACCGGAGGAA

E1A R: TCACCCACTGCCCATAATTT

E1A probe: GGACCTGTGGCATGTTTGTCTACA

G6PD-F: TGAATGATGCAGCTCTGATCC

G6PD-R: CACGATGATGCGGTTCCA

G6PD probe: GTAACGCAGCTCCGGGCT

Primers for E1A gene cloning

E1A289-F: CGGCGGTACCATGAGACATATTATCTGCCACG

E1A289-R: GCGCCCTCGAGTGGCCTGGGGCGTTTACAG

Oligonucleotides for Hsp90 mutagenesis

HSP90αE46A-F: GAGATTTTCCTTCGGGCGTTGATCTCTAATGCT

HSP90αE46A-R: AGCATTAGAGATCAACGCCCGAAGGAAAATCTC
